# A Case of New-Onset Dermatomyositis in the Second Trimester of Pregnancy: A Case Report and Review of the Literature

**DOI:** 10.1155/2016/6430156

**Published:** 2016-07-10

**Authors:** Tayfun Akalin, Hatice Akkaya, Barış Büke, İbrahim Koçak

**Affiliations:** ^1^Department of Internal Medicine, Section of Rheumatology, Kayseri Training and Research Hospital, 38020 Kayseri, Turkey; ^2^Department of Obstetrics and Gynecology, Kayseri Training and Research Hospital, 38020 Kayseri, Turkey; ^3^Department of Internal Medicine, Kayseri Training and Research Hospital, 38020 Kayseri, Turkey

## Abstract

Dermatomyositis (DM), a subtype of idiopathic inflammatory myopathies (IIMs), is characterized by skin rash, proximal muscle weakness, and inflammatory infiltrates in the muscle tissue. The peak incidence of the disease is at the age of 50–60 years, and only 14% of the patients with IIMs are estimated to present during reproductive years. Because of the limited pregnancy experience in patients with IIMs, little is known regarding the effects of DM on pregnancy or vice versa. We herein report a 40-year-old woman who developed DM in the second trimester of her pregnancy and did not respond to treatment with methylprednisolone. Her pregnancy was terminated at the 32nd week of gestation, due to preeclampsia and fetal distress. She delivered a healthy baby and improved rapidly after delivery. We have searched PubMed for relevant articles and reviewed previously published cases.

## 1. Introduction

Dermatomyositis (DM) is an idiopathic inflammatory myopathy (IIM) characterized by skin rash, proximal muscle weakness, and inflammatory infiltrates in the muscle tissue. It is a rare disease with an estimated prevalence of 11 cases per 100,000 individuals [1]. Although it may begin at any age, the peak incidence is at the age of 50–60 years. Only 14% of patients with IIMs are estimated to present during childbearing ages [2]. Therefore, pregnancy-associated cases are rare in the literature.

Little is known regarding the effects of DM on pregnancy or vice versa. Case reports and small series indicate that most patients with quiescent disease at conception remain inactive during pregnancy and have good pregnancy outcomes [3, 4], whereas preexisting active disease or onset of DM during pregnancy was reported to be associated with high frequency of fetal death and premature delivery [4]. Contrary to systemic lupus erythematosus (SLE), DM seems to remain inactive during pregnancy in most patients [3–5]. There are conflicting data as to whether pregnancy is a triggering factor for the development of DM [3, 6]. Although corticosteroids are the mainstay of treatment and effective in most patients, some patients with DM are nonresponsive or intolerant to corticosteroids and treatment options in these patients are not clear.

We herein report a 40-year-old woman who developed DM in the second trimester of her pregnancy and did not respond to treatment with methylprednisolone. Her pregnancy was terminated at the 32nd week of gestation due to preeclampsia and fetal distress. We have searched PubMed using the search terms “pregnancy”, “dermatomyositis”, and “idiopathic inflammatory myopathy” and reviewed relevant articles.

## 2. Case Report

**Figure 1:**
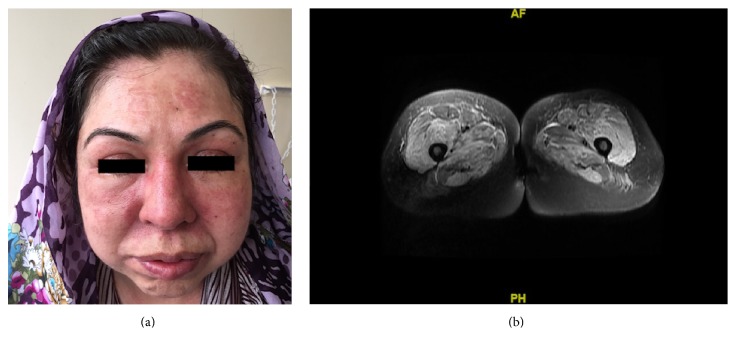
Skin and muscle involvement: (a) rash on her face and (b) bilateral diffuse inflammation in thigh muscles (magnetic resonance imaging).

A 40-year-old woman in the 27th week of her fourth pregnancy was referred to our rheumatology clinic because of a three-week history of a skin rash, arthralgia, and weak positive anti-nuclear antibody test by immunofluorescence assay (ANA-IFA). Her previous three pregnancies were uneventful. She did not have a history of recent infections nor a potential exposure to toxic or medical agents. Physical examination on admission revealed bilateral periorbital edema and erythema on her eyelids (heliotrope rash) as well as diffuse rash on the face (Figure 1(a)). She had bilateral erythematous macules on the extensor surfaces of the metacarpophalangeal and proximal interphalangeal joints (Gottron's sign). There was also symmetrical and proximal muscle weakness in the upper and lower extremities. Other physical examination findings were unremarkable. Laboratory findings were as follows: serum creatine kinase (CK): 2138 U/L (normal: 29–200), lactate dehydrogenase: 520 U/L (normal: 140–280), aspartate aminotransferase: 113 U/L (normal: <35), and alanine aminotransferase: 58 U/L (normal: <35). ANA-IFA was weakly positive at 1/100 titration (homogenous pattern). Anti-Ro52 was positive, but antibodies to Jo-1 or other extractable nuclear antigens were negative. Serum anti-double stranded DNA, rheumatoid factor, and complement levels were within normal ranges. Serum creatinine level and urine analysis were normal. Erythrocyte sedimentation rate was 31 mm/h and serum C-reactive protein level was 17.9 mg/L (normal 0–5). Electromyography of the right deltoid muscle revealed fibrillations and small polyphasic motor unit action potentials. Magnetic resonance imaging showed diffuse edema in thigh muscles (Figure 1(b)). She declined muscle biopsy. Investigations for occult malignancies including breast ultrasound, pelvic and abdominal ultrasound, peripheral blood smear, and fecal occult blood test were all negative. Serum CA-125 level was 6.5 U/mL (normal 0–35). She was diagnosed with dermatomyositis according to Bohan and Peter's criteria [27, 28] and started on methylprednisolone 32 mg/day orally. Five weeks later, however, no improvement was noted in muscle strength, skin rash, or serum CK level. Her blood pressure was 180/120 mmHg and she had developed bilateral pretibial edema. She was diagnosed as having preeclampsia and her pregnancy was terminated by an emergency caesarean section at the 32nd week of the gestation. She gave birth to a 1800 g male infant with no apparent congenital malformation. In postpartum period, we searched the mother for the presence of anti-phospholipid antibodies. Anti-cardiolipin and anti-*β*2 glycoprotein-1 antibodies (immunoglobulin G and M isotypes) were found to be negative. Lupus anticoagulant test could not be performed due to technical limitations.

After delivery, her muscle strength and rash improved rapidly. The dosage of methylprednisolone was tapered off. At postpartum 6th week, she was on methylprednisolone 8 mg/day and serum CK levels were within normal limits. There was only mild facial rash left, but heliotrope rash and Gottron's sign had completely resolved.

## 3. Discussion

DM in pregnant patients may be present before the onset of pregnancy, occur during pregnancy, or develop in postpartum period [4].  Table 1 summarizes the pregnancies with DM [2–4, 6–26]. There are 53 pregnancies in 41 patients. The onset of DM was before pregnancy in 21 patients, during pregnancy in 16, and in puerperal period in 4.

Gutierrez et al. [6] reported 18 female patients with IIMs (2 polymyositis-PM, 16 DM). Four of these patients (22%) had developed DM/PM in pregnancy-related period (3 in pregnancy, 1 in postpartum period), and they speculated that pregnancy was a precipitating factor in the onset of inflammatory myositis. However, a recent retrospective cohort study [3] with 41 patients with DM showed that only in one patient (2.4%) did the disease begin in the pregnancy-related period (i.e., during pregnancy or in puerperal period). Furthermore, the observed and expected proportions of IIM patients with the onset of disease in pregnancy-related period were similar (3.9% versus 3.7%, resp.). In a case series with 78 female patients with DM, no patient was identified with the onset of her disease during pregnancy or in postpartum period [10]. Moreover, in another case series with 173 female patients with DM/PM, only one patient had disease onset during pregnancy [22]. It seems that pregnancy is not a triggering factor for the development of DM.

Pregnancy outcomes in DM are shown in Table 2 (derived from Table 1). There are 6 (11.1%) premature babies, 11 (20.4%) abortions, and 3 (5.6%) intrauterine growth restrictions (IUGR) in 53 pregnancies (1 pair of twins). Patients with active disease have more frequent preterm birth compared to inactive patients (23.8% versus 3%, *p* = 0.028). Relative frequencies of total fetal loss and IUGR in patients with active disease are similar to those in inactive patients (28.6% versus 24.2% and 4.8% versus 6.1%, resp.).

It is controversial whether pregnancy is a risk factor for exacerbation of DM. In a case series, exacerbation occurred in 3 of 7 pregnancies (43%) and pregnancy was considered as a precipitating factor for DM activation [6]. In another case series, however, no exacerbation was observed in 11 pregnancies; DM improved in 8 pregnancies and remained inactive in the other three [3]. Review of the published cases indicates only four DM exacerbations [6, 10, 26] in 37 pregnancies (10.8%). This flare rate is markedly less frequent than that reported in pregnant patients with SLE (57%) [5].

Our case did not respond to moderate dose steroid during pregnancy. Nonresponsiveness to corticosteroid treatment during pregnancy and rapid improvement after delivery suggest that pregnancy-related factors such as hormonal changes or fetal antigen transfer to the mother during pregnancy may have been involved in the pathogenesis in this patient [29]. However, it should be noted that, in addition to pregnancy-related factors, inadequate steroid dose could also be responsible for treatment failure in our patient.

Corticosteroids are relatively safe drugs during pregnancy and are the first choice of treatment in pregnant patients with DM. However, some patients are nonresponsive or intolerant to corticosteroids [20]. Treatment options in this group of patients are not clear. Four patients were treated with IVIG either during pregnancy [20, 25] or in postpartum period [19, 24]. IVIG treatments were successful in all patients. Babies were born at term and healthy. No patient was treated with other immunosuppressive drugs such as azathioprine or cyclosporine. Inadvertent methotrexate use resulted in abortions in two patients [3, 4].

In conclusion, pregnancy does not seem to be a triggering factor for the development of DM. In most patients, DM improves or remains inactive during pregnancy. Active disease and/or its treatment may be associated with preterm birth and/or preeclampsia. IVIG should be considered as a therapeutic option in steroid resistant/intolerant pregnant patients, especially if delivery is not a feasible option.

## Figures and Tables

**Table 1:** Dermatomyositis complicating pregnancy. Review of the cases.

Author [ref.]	Patient	Age at diagnosis (years)	DM onset	Disease activity (during pregnancy)	Treatment	Fetal outcome
Glickman [7]	1	27	BP	Improved	Prednisone	Healthy

Masse [8]	1	26	BP	Improved	ACTH	Healthy
				Inactive	ACTH	Abortion

Tsai et al. [9]	1	33	DP	Active	No treatment	Neonatal death

Gutierrez et al. [6]	1	NI	DP	Active	Prednisone	Abortion
	2	NI	DP	Active	Prednisone	Stillbirth
	3	JDM	BP	Inactive	No treatment	Abortion
				Exacerbation	Prednisone	Premature
	4	NI	PP	Inactive	No treatment	Abortion
				Inactive	No treatment	Caesarean at term
	5	NI	BP	Inactive	No treatment	Healthy
	6	NI	BP	Exacerbation	Prednisone	Premature
				Inactive	No treatment	Twins Premature/neonatal death

King and Chow [10]	1	12	BP	Inactive	No treatment	IUGR
				Inactive	No treatment	Healthy
	2	20	BP	Inactive	No treatment	Healthy
				Inactive	No treatment	Healthy
	3	25	BP	Exacerbation	Prednisone	Healthy

England et al. [11]	1	35	DP	Active	Steroid	IUGR

Ishii et al. [12]	1	31	DP	Active	Steroid	Healthy
				Inactive	Steroid	Healthy

Pinheiro Gda et al. [2]	1	14	DP	Active	Steroid	Healthy

Suwa et al. [13]	1	29	PP	—	Spontaneous remission	—

Harris et al. [14]	1	29	DP	Active	Induced labor, pulse steroid PP	Healthy

Solomon and D'Alton [15]	1	28	DP	Active	Steroid	Healthy

Kofteridis et al. [16]	1	25	DP	Active	Pulse steroid	Abortion

Kanoh et al. [17]	1	33	PP	—	Prednisolone PP	—

Lee and Yoo [18]	1	33	PP	—	Methylprednisolone PP	—

Silva et al. [4]	1	22	BP	Active, no change	Steroid, MTX	Abortion
	2	28	BP	Inactive	Prednisolone	Healthy

Park et al. [19]	1	22	DP	Active	Therapeutic abortion, IVIG PP	Abortion

Mosca et al. [20]	1	32	DP	Active	Methylprednisolone, IVIG	Healthy

Pasrija et al. [21]	1	27	DP	Active	Dexamethasone, HCQ	Healthy

Váncsa et al. [22]	1	22	BP	Inactive	Methylprednisolone	Healthy
				Inactive	Methylprednisolone	Abortion
	2	28	BP	Inactive	Methylprednisolone	Healthy
	3	33	BP	Inactive	No treatment	Healthy
	4	37	DP	Active	Methylprednisolone	Premature
	5	30	BP	Inactive	No treatment	Healthy

Chopra et al. [23]	1	28	BP	Inactive	No treatment	Healthy
				Inactive	No treatment	IUGR

Nozaki et al. [24]	1	31	DP	Active	Prednisolone, IVIG PP	Premature

Linardaki et al. [25]	1	42	DP	Active	Methylprednisolone, IVIG	Healthy

Madu et al. [26]	1	JDM	BP	Exacerbation	Prednisolone	Healthy

Pinal-Fernandez et al. [3]	1	35	BP	Improvement	Prednisone, MTX	Induced abortion
				Improvement	Prednisone	Healthy
				Improvement	Prednisone	Healthy
	2	35	BP	Improvement	Prednisone	Healthy
				Improvement	Prednisone	Healthy
	3	38	BP	Improvement	Prednisone	Induced abortion (anencephaly)
				Improvement	Prednisone	Healthy
				Improvement	Prednisone	Healthy
	4	32	PP	—	Prednisone PP	—
	5	31	BP	Asymptomatic	No treatment	Healthy
				Asymptomatic	No treatment	Abortion
	6	36	BP	No change	Prednisone	Healthy

Present case	1	40	DP	Active	Methylprednisolone	Premature

BP, before pregnancy; DP, during pregnancy; PP, puerperal period; NI, not indicated; JDM, juvenile dermatomyositis; ACTH, adrenocorticotrophic hormone; IVIG, intravenous immunoglobulin; MTX, methotrexate; HCQ, hydroxychloroquine; IUGR, intrauterine growth restriction.

**Table 2:** Pregnancy outcomes in active and inactive DM.

Pregnancy outcomes	Disease activity during pregnancy		*p* value^*∗*^	OR (95% CI)
	Active (*n* = 21) (%)	Inactive (*n* = 32) (%) (1 pair of twins)		
Healthy	9 (42.8)	22 (66.7)	NS	0.38 (0.12–1.16)
Premature birth	5 (23.8)	1 (3.0)	0.028	10.00 (1.08–92.94)
IUGR	1 (4.8)	2 (6.1)	NS	0.78 (0.07–9.12)
Abortion	4 (19)	7 (21.2)	NS	0.87 (0.22–3.44)
Stillbirth	1 (4.8)	0 (0)	NS	—
Neonatal death	1 (4.8)	1 (3.0)	NS	1.60 (0.10–27.04)
Total fetal loss^*∗∗*^	6 (28.6)	8 (24.2)	NS	1.25 (0.36–4.31)

^*∗*^Chi-square or Fisher's exact test used where appropriate. *p* < 0.05 is considered to be statistically significant.

^*∗∗*^Sum of the abortions, stillbirths, and neonatal deaths.

DM, dermatomyositis; OR, odds ratio; NS, not significant; IUGR, intrauterine growth restriction.
